# Supplementary material for: A survey of the sperm whale (Physeter catodon) commensal microbiome
Source: PeerJ. 2019 Jul 4;7:e7257. doi: 10.7717/peerj.7257 (PMC6612419; doi:10.7717/peerj.7257)
Supplement: Table S1 [file peerj-07-7257-s001.docx]

**Table S1. Information of marine mammals’ gut microbiome data.**

| **Spices Name** | **Sample ID** | **Database** | **Accession ID** |
| --- | --- | --- | --- |
| *Delphinapterus leucas* | BelugaWhale.1 | EMBI | mgm4683145 |
| *Delphinapterus leucas* | BelugaWhale.2 | EMBI | mgm4683159 |
| *Tursiops truncatus* | Dolphin.1 | NCBI | SRR597472 |
| *Tursiops truncatus* | Dolphin.2 | NCBI | SRR597498 |
| *Tursiops truncatus* | Dolphin.2 | NCBI | SRR597499 |
| *Tursiops truncatus* | Dolphin.3 | NCBI | SRR597502 |
| *Tursiops truncatus* | Dolphin.4 | NCBI | SRR597660 |
| *Tursiops truncatus* | Dolphin.5 | NCBI | SRR597705 |
| *Tursiops truncatus* | Dolphin.6 | NCBI | SRR597663 |
| *Florida manatee* | Manatee.1 | NCBI | SRR2960000 |
| *Florida manatee* | Manatee.2 | NCBI | SRR2960045 |
| *Florida manatee* | Manatee.3 | NCBI | SRR2960046 |
| *Florida manatee* | Manatee.4 | NCBI | SRR2960044 |
| *Megaptera novaeangliae* | HumpbackWhale.1 | EMBI | mgm4683131 |
| *Megaptera novaeangliae* | HumpbackWhale.2 | EMBI | mgm4683151 |
| *Megaptera novaeangliae* | HumpbackWhale.3 | EMBI | mgm4683156 |
| *Balaenoptera borealis* | SeiWhale | EMBI | mgm4683165 |
| *Eubalaena glacialis* | RightWhale.1 | EMBI | mgm4683135 |
| *Eubalaena glacialis* | RightWhale.2 | EMBI | mgm4683138 |
| *Eubalaena glacialis* | RightWhale.3 | EMBI | mgm4683160 |
| *Eubalaena glacialis* | RightWhale.4 | EMBI | mgm4683161 |
| *Eubalaena glacialis* | RightWhale.5 | EMBI | mgm4683164 |
| *Eubalaena glacialis* | RightWhale.6 | EMBI | mgm4683167 |
