# Supplementary material for: A survey of the sperm whale (Physeter catodon) commensal microbiome
Source: PeerJ. 2019 Jul 4;7:e7257. doi: 10.7717/peerj.7257 (PMC6612419; doi:10.7717/peerj.7257)
Supplement: Table S2 [file peerj-07-7257-s002.docx]

**Table S2.** **Summary of metagenomics sequence data for sperm whale.**

| **Sample** | **Read length (bp)** | **Raw data (bp)** | **Clean data (bp)** | **Removed host data (bp)** | |
| --- | --- | --- | --- | --- | --- |
| Blood | 100_100 | 13,556,461,600 | 19,452,596,600 | 16,048,023,000 |  |
| Fecal | 100_100 | 97,235,769,000 | 67,708,034,400 | 52,632,121,000 |  |
| Muscle | 100_100 | 260,551,892,400 | 215,933,825,400 | 14,581,691,200 |  |
