# Supplementary material for: A survey of the sperm whale (Physeter catodon) commensal microbiome
Source: PeerJ. 2019 Jul 4;7:e7257. doi: 10.7717/peerj.7257 (PMC6612419; doi:10.7717/peerj.7257)
Supplement: Table S3 — Phyla level appears in this table. [file peerj-07-7257-s003.docx]

**Table S3. The abundance of the microbial species which were detectable in all three tissues.**

| **Taxnomy** | **blood** | **fecal** | **muscle** |
| --- | --- | --- | --- |
| Tenericutes | 0.00018 | 2.18E-05 | 1.06E-06 |
| Bacteroidetes | 0.00050 | 0.00041 | 1.36E-06 |
| Proteobacteria | 0.00175 | 0.00035 | 5.86E-05 |
| Fusobacteria | 0.00132 | 0.00033 | 2.59E-06 |
| Spirochaetes | 0.00060 | 0.00088 | 3.16E-06 |
| Deferribacteres | 0.00016 | 1.82E-05 | 8.22E-07 |
| Firmicutes | 0.62037 | 0.00691 | 0.00099 |
| Other | 0.37513 | 0.99108 | 0.99894 |

Note：Phyla level was shown in this table.
