# Supplementary material for: A survey of the sperm whale (Physeter catodon) commensal microbiome
Source: PeerJ. 2019 Jul 4;7:e7257. doi: 10.7717/peerj.7257 (PMC6612419; doi:10.7717/peerj.7257)
Supplement: Figure S1 [file peerj-07-7257-s005.pdf]

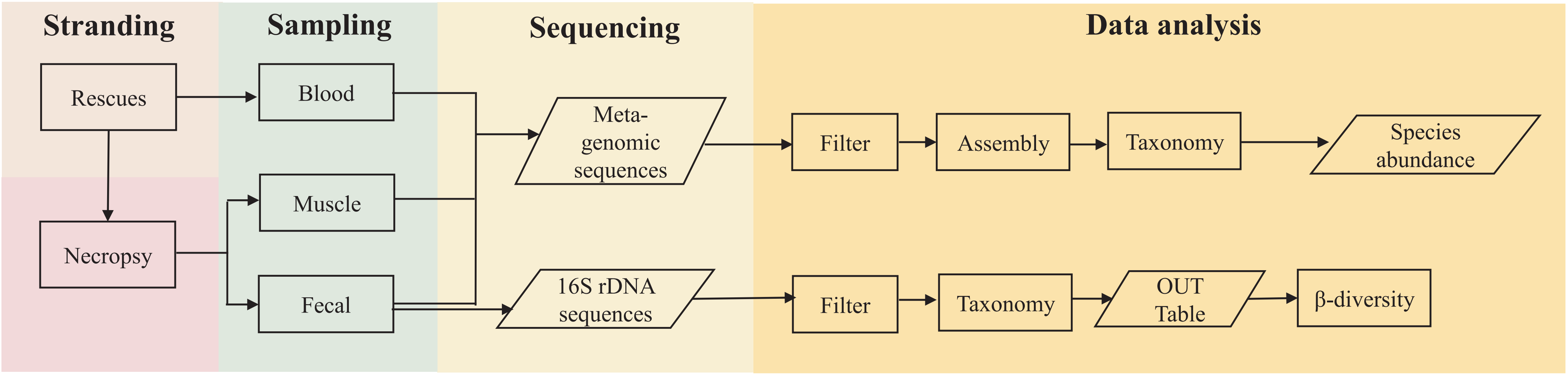
Stranding
Sampling
Sequencing
Data analysis
Rescues
Blood
Meta-
genomic
sequences
Filter
Assembly
Taxonomy
Species
abundance
Muscle
Necropsy
Fecal
16S rDNA
sequences
Filter
Taxonomy
OUT
Table
β-diversity
