# Supplementary figures and images for: A survey of the sperm whale (Physeter catodon) commensal microbiome
Source: PeerJ. 2019 Jul 4;7:e7257. doi: 10.7717/peerj.7257 (PMC6612419; doi:10.7717/peerj.7257)

M

B

S

1500

1000

900

800

700

600

500

400

300

200

100

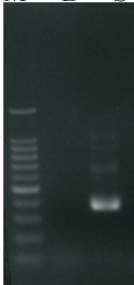

Supplement: Figure S2 — M is the marker, B is the negative control, S is 16S V4 library. [file peerj-07-7257-s006.pdf]

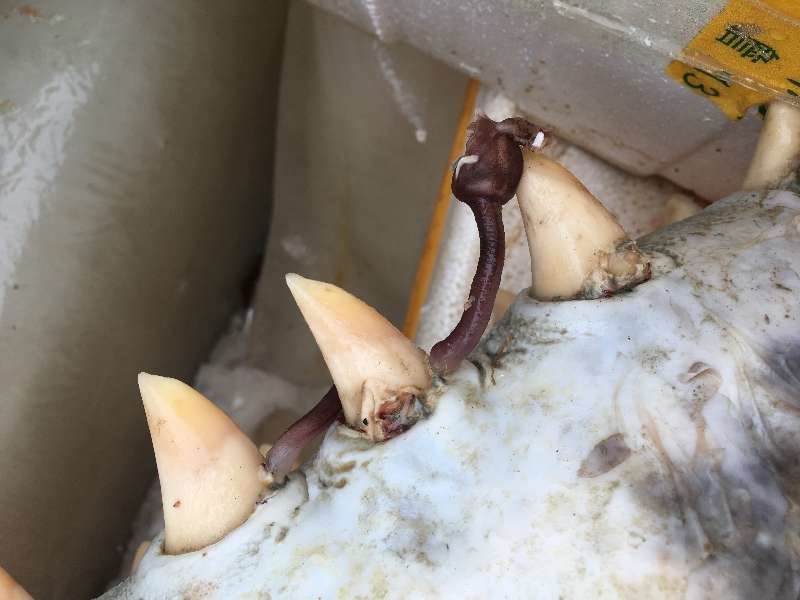

Supplement: Figure S3 [file peerj-07-7257-s007.jpg]
